# Influence of ozone on the rheological and electrical properties of stored human blood

**DOI:** 10.7555/JBR.26.20110070

**Published:** 2012-05-17

**Authors:** Hoda El-Saied Abdel Baieth, Islam Shoukri Elashmawi

**Affiliations:** a Department of Physics, Faculty of Science, Benha University, Benha 13518, Egypt;; b Department of Spectroscopy, Physics Division, National Research Center, Giza 12311, Egypt;; c Department of Physics, Faculty of Science, Taibah University, Al-Ula 100823, Saudia Arabia.

**Keywords:** blood, ozone generation, alternate current conductivity, viscosity

## Abstract

Blood stored in a blood bank undergoes a series of chemical changes and storage lesions. The purpose of this study was to assess the effect of ozone on the rheological and electrical properties of stored human blood. Venous blood samples, obtained from three healthy humans, were treated with different concentrations of ozone (30, 50, 70 and 80 µg/mL) for three weeks *in vitro*. Ozone was generated from portable medical-grade oxygen using electrical corona arc discharge. The ultraviolet-visible absorption of hemoglobin in the wavelength of 300-700 nm showed that ozone in this range did not interact with iron ions and it was not toxic below the concentration of 80 µg/mL. The changes of blood viscosity were also measured. The electrical conductivity and permittivity, in the frequency range from 5 to 50 MHz, were measured in the control and treated samples subjected to different concentrations of ozone at different stored periods. The results showed that the conductivity and permittivity measurements may serve as a useful indicator in the quality assessment of blood samples stored in the blood bank.

## INTRODUCTION

Blood stored in the blood bank undergoes a series of chemical changes and storage lesions. The decrease in the viability of red blood cells (RBCs) following transfusion, which is defined as the percentage of cells remaining in the circulation of the recipient 24 h after transfusion, is considered a consequence of storage lesions[1],[2]. Zhao *et al*.[1] observed that chemical changes in RBCs, due to alterations in the concentrations of Na^+^, K^+^ and Cl^-^ and adenosine triphosphate (ATP), affect the electrical impedance of blood. In addition, Berezina *et al.*[3] found that significant changes of RBC shape and deformability in stored blood starts at the second week of storage and progresses during the rest of the storage period, accompanied by a progressive increase in hemolysis and acidosis.

The importance of ozone in the biological systems is due to the interaction of ozone with blood constituents. Ozone is a molecule that has a molecular weight of about 48, a density of one-and-a-half times that of oxygen and contains a large excess of energy (143 kJ/mol). It has a bond angle of 127, which resonates among several forms. As early as the First World War, the bactericidal properties of ozone were used to treat infected wounds. In the last few years, increasing interest in ozone treatment has progressed rapidly, especially in its effect on biological systems and chemical applications.

Travagli *et al*.[4] observed that when erythrocytes were exposed to therapeutic doses of ozone (10-80 µg/mL), neither peroxidation of membrane phospholipids nor generation of methemoglobin were present. How hydrogen peroxide acts on the different blood cells has been investigated[5]-[7]. The reactive oxygen species (ROS) molecules are able to act as an ozone responsible for eliciting several biological and therapeutic effects[8],[9]. Ozone leads to the destruction of both viruses and bacteria[10],[11]. A sufficient amount of ozone breaks through the cell membrane, which leads to the destruction of bacteria. In addition, ozone destroys viruses by diffusing through the protein coat into the nucleus acid core, resulting in damage of viral RNA. Thus, ozone is considered to act as a mild enhancer of the immune system by activating neutrophils and stimulating the synthesis of some cytokines[12],[13].

H_2_O_2_ crosses the cell membrane and activates genes that regulate the transcription of RNAs of several cytokines[9],[12]. Ozone induces the release of cytokines by leucocytes. Ozone therapy was found to be an effective, safe and less expensive method in patients with hepatitis C. In Egypt, hepatitis C is a major medical problem and the treatment is very expensive. It is postulated that more than 15%, i.e more than 10 million of the population in Egypt, suffer from hepatitis C[13].

In the present study, we measured the ultraviolet (UV)-visible spectrum and dielectric dispersion of hemoglobin and examined the whole conductivity range up to physiological value. Hemoglobin is considered as a model system for human erythrocytes because it has no compartmental structure. Dielectric dispersion of hemoglobin solutions can be observed as a decrease of the permittivity—the dielectric decrement[14]. Dielectric properties of hemoglobin are important for their physiological functions, such as interaction with charged ligands. Such an effect could be interpreted in terms of a permanent dipole moment[15]. To calculate the electric dipole moment, the distribution of fixed charges on hemoglobin as well as its core bond moment was considered and the proton fluctuations were considered as significant contribution to the observed dipole moment[16].

## MATERIALS AND METHODS

### Ozone generation

Ozone was generated from portable medical-grade oxygen using electrical corona arc discharge (Rastatt, Hansller GmbH, Germany). Ozone concentration was controlled in real-time by photometric determination as recommended by the Standardization Committee of the International Ozone Association (IOA).

### Collection of human blood samples

The protocol was approved by the local institutional review board at the authors' affiliated institutions. The blood samples were obtained from the veins of three 21-year-old healthy male non-smoking volunteers, who did not take any medications. They provided informed consent for the study. The citrate phosphate dextrose (CPD) human blood preserve consisted of approximately 70 mL CPD stabilizer and 450-550 mL human blood. To investigate the storage-dependent changes of blood in separate baby bags, blood was divided into 5 bags and ozonized at concentrations of 30, 50, 70 and 80 µg/mL within 6 h after blood collection. For each sample, complete blood count was done to measure hemoglobin concentration. Hemoglobin was extracted and diluted with 0.1 mol/L phosphate buffer (pH 7.4).

### Gas delivery

Gas delivery was carried out with a single dose of ozone (concentration per volume) as follows: a predetermined volume of a gas mixture composed of oxygen (95%-99%) and ozone (1%-5%), at ozone concentrations of 30, 50, 70 and 80 µg/mL, was collected with a syringe and immediately introduced into the bags containing the blood samples. Ozone reacted completely with substrates within 10 min and the blood samples reacted with the ozone dose totally. The final gas pressure remained at normal atmospheric pressure. Blood samples without ozone were used as control. All analyses for the blood samples were carried out in the same day and after 7, 14 and 21 d.

### Spectroscopic determination

The absorbance of hemoglobin was determined using the JASCO V-530 UV-visible spectrophotometer (Tokyo, Japan) equipped with 10-mm quartz cells. The absorption spectra were obtained over the range of 300 to 700 nm at room temperature. A data interval of 1 nm was selected, with a single average time of 0.5 s for each data point. Before any determinations, samples were centrifuged at 3000 *g* for 20 min and diluted, and the erythrocytes were washed with saline.

### Viscosity evaluation

The Brookfield DV-III Programmable Cone and Plate Rheometer was used to measure the blood parameters including shear stress and viscosity under controlled conditions of shear rates, temperature and time within the range of 0.4-1000 mPas. The Bohlin Programmable Rheometer Model (Bohlin Instruments Ltd, UK) was applied to measure the blood parameters of shear stress and viscosity under controlled conditions of shear rates, temperature and time. The sample volume required for this experiment was 0.5 mL fresh heparinized blood. The computer-controlled measurement protocols allowed ease of operation and reproducible measurement conditions. The shear rate of a given measurement was determined by the rotational speed, size and shape of the spindle, and the size and shape of the container used, as well as the distance between the container wall and the spindle surface. Temperature was controlled accurately at 37.0±0.1°C during the experiments.

### Electrical properties of blood

Hemoglobin solution was prepared according to the method of Trivelli *et al*.[14]. Dielectric measurements were done on the collected hemoglobin from all groups in the frequency range from 50 Hz to 5 GHz using the Chen Hwa 1061 LCZ meter manufactured by Taiwan IEEE-488 interface, with a conductivity cell type PW 950/60 manufactured by Philips, Holland. The cell has two parallel squared back electrodes of 0.8 cm side each and area 0.64 cm^2^ (A), and 1 cm separation distance (d). Dielectric measurements were carried out by measuring the capacitance (C) and the resistance (R), which were used to calculate the real (έ) and imaginary (ϵ″) parts of the complex permittivity and the conductivity (σ) using the following equations. 
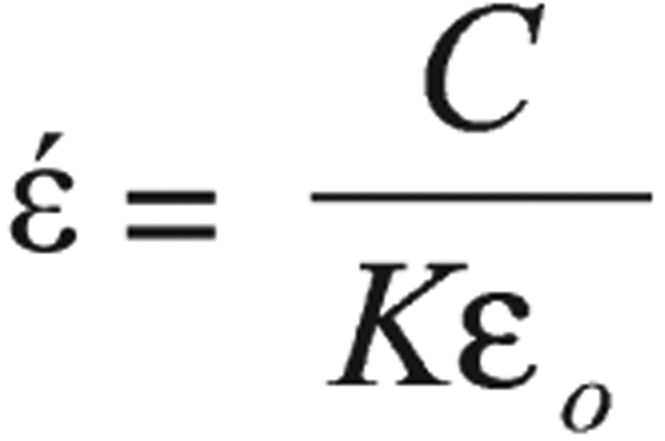
(1)

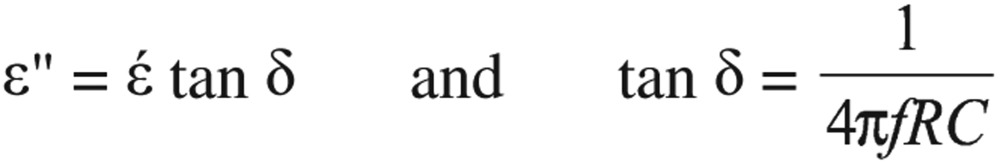
(2)

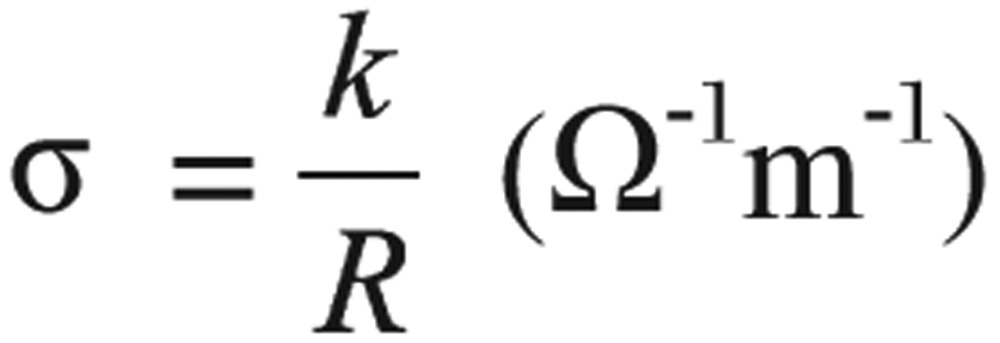
(3)

where *K* is the cell constant, which is a function of cell dimensions, and (ϵ_o_) is the permittivity of free space. Relaxation time (τ) can be calculated from the relation[17]: 
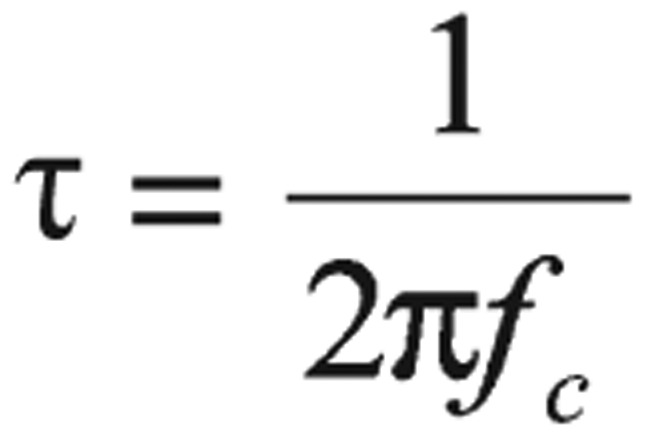
(4)

where *f*_c_ is the critical value of the frequency that corresponds to the midpoint of the dispersion curve.

The AC electrical conductivity of all the prepared samples at room temperature was calculated over a frequency range from 50 Hz to 5 MHz by using the following formula[17],[18]: 
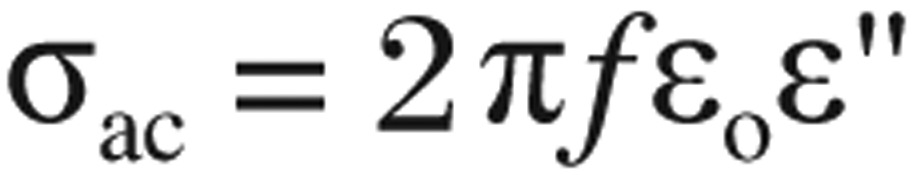
(5)

where *f* is frequency, ϵ_o_ is the permittivity of free space (8.854×10^−12^ F/m), and ϵ″ is the imaginary dielectric constant (dielectric loss).

### Statistical analysis

The results were presented as mean±SD, and all statistical analyses were performed using the statistical software SPSS version 14.0 (SPSS Inc., Chicago, IL,USA). Analysis of variance (ANOVA) was used to test the statistical significance of changes in blood parameters at different storage times. A *P*value < 0.01 was considered statistically significant.

## RESULTS

### Influence of ozone on the optical spectra of hemoglobin

**Fig. 1:**
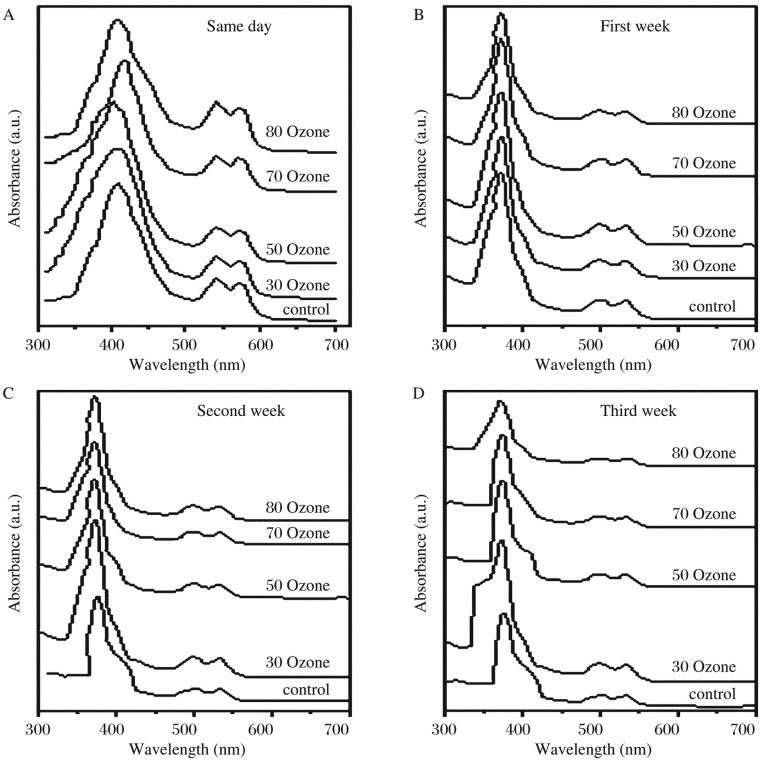
Absorption spectra of ozone-treated hemoglobin. The absorption spectra of hemoglobin for all blood samples in the same day (A) and after 7 (B), 14 (C) and 21 d (D) with different concentrations of ozone (control, 30, 50, 70 and 80 ug/mL) in the range of 300 to 700 nm at room temperature.

**Fig. 2:**
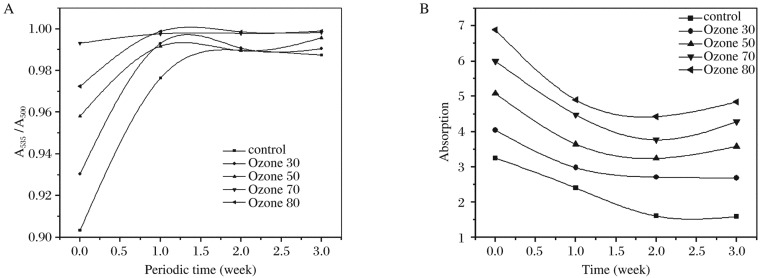
Relative and time-dependent absorbance of ozone treated blood samples. A: Relative absorbance. Blood samples were treated with different concentrations of ozone (control, 30, 50, 70 and 80 µg/mL) and absorbance was read at 500 and 535 nm. B: Time-dependent absorbance. Blood samples were treated with different concentrations of ozone (control, 30, 50, 70 and 80 µg/mL) and absorbance was read at 410 nm for the indicated time points.

The absorption spectra of hemoglobin for all blood samples treated with different concentrations of ozone (control, 30, 50, 70 and 80 µg/mL) in the same day and after 7, 14 and 21 d in the range from 300 to 700 nm are shown in ***Fig. 1***. The optical absorption spectra of hemoglobin for the samples obtained in the same day showed three bands. Two characteristic bands at 570 and 540 nm were attributed to oxyhemoglobin, and the band at 410 nm was assigned to hem-hem interaction. Those bands shifted to the lower wavelength of 371, 500 and 535 nm after 7, 14 and 21 d, respectively. Hematological data in all groups are shown in ***Table 1***. The results showed a significant decrease in hemoglobin with the increase in ozone concentration, while the mean corpuscular volume (MCV) increased to about 35.6% in the control blood stored without ozone treatment. In addition, a significant increase in hematocrit percentage (HCT%) was observed in the stored blood, which was not dependent on the ozone concentration. ***Fig. 2A*** shows the ratio of the absorbance at 535 and 500 nm for all samples. There was a significant increase in the absorbance values for samples treated with ozone at concentrations of 30, 50 and 80 µg/mL in the same day, and there was no significant change observed when they were treated with ozone at a concentration of 70 µg/mL. The conversion of oxyhemoglobin to methemoglobin was not observed in all cases. ***Fig. 2B*** demonstrates significant reductions in the absorbance values at 410 nm, corresponding to the hem-hem interaction, and the absorbance value gradually decreased with time in the control, 50 and 80 µg/mL of the ozone groups compared with those treated with 30 and 70 µg/mL.

**Table 1:** Hematological data in all groups

Hematological parameter	Storage period	Control	Ozone (30 ug/mL)	Ozone (50 ug/mL)	Ozone (70 ug/mL)	Ozone (80 ug/mL)
Hb (gm/dL)	Same day	14.20 ± 0.01	13.65 ± 0.06	11.65 ± 0.02	12.20 ± 0.02	8.90 ± 0.04
	First week	12.20 ± 0.01	11.65 ± 0.06	11.15 ± 0.02	11.00 ± 0.02	8.00 ± 0.04
	Second week	11.20 ± 0.01	11.90 ± 0.06	11.05 ± 0.02	11.90 ± 0.02	8.60 ± 0.04
	Third week	9.20 ± 0.01	11.65 ± 0.06	11.00 ± 0.02	12.50 ± 0.02	7.90 ± 0.04
HCT (%)	Same day	37 20 ± 1.10	38.20 ± 0.09	37 50 ± 0.05	37.90 ± 0.05	41.10 ± 0.06
	First week	38.20 ± 1.10	39 20 ± 1.10	37.90 ± 1.10	38.10 ± 0.01	37.20 ± 0.01
	Second week	39 20 ± 1.10	37 20 ± 1.10	37.70 ± 1.10	38.20 ± 0.01	37 20 ± 0.01
	Third week	41.20 ± 1.10	37 20 ± 1.10	38.20 ± 1.10	38.50 ± 0.01	37 20 ± 0.01
MCV (f)	Same day	66.90 ± 0.01	77.04 ± 0.04	79.40 ± 0.01	78.51 ± 0.01	79.30 ± 0.03
	First week	83.90 ± 0.01	76.04 ± 0.04	77.04 ± 0.04	76.04 ± 0.04	81.04 ± 0.04
	Second week	89.90 ± 0.01	79.04 ± 0.04	76.04 ± 0.04	76.09 ± 0.04	83.04 ± 0.04
	Third week	90.90 ± 0.01	79.04 ± 0.04	79.04 ± 0.04	80.04 ± 0.04	87.04 ± 0.04

Hb: hemoglobin; HCT: hematocrit; MCV: mean corpuscle volume.

### Influence of ozone on viscosity

**Fig. 3:**
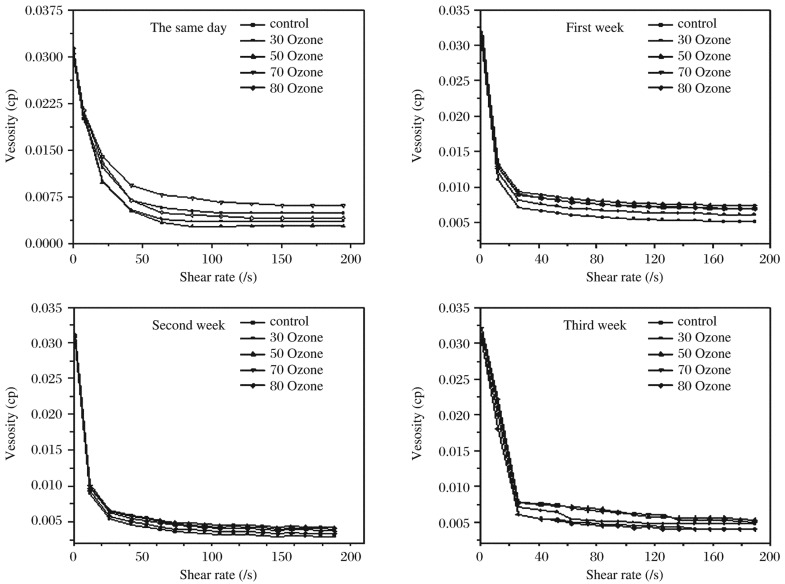
The shear rate is dependent on blood viscosity in all blood samples treated with ozone at concentrations of 30, 50, 70 and 80 µg/mL in the same day and after 7, 14 and 21 d.

***Fig. 3*** shows the shear rate-dependent blood viscosity in all blood samples treated with ozone at concentrations of 30, 50, 70 and 80 µg/mL in the same day and after 7, 14 and 21 d. ***Table 2*** demonstrates that blood viscosity changed with ozone concentrations and over time. It was observed that blood viscosity varied following treatment with ozone at concentrations of 30, 50, 70 and 80 µg/mL, and the viscosity of the blood stored without ozone treatment increased. For blood samples treated with different concentrations of ozone, there were significant decreases in viscosity.

**Table 2:** Changes of blood viscosity with ozone at different concentrations and time points

Group	Same day	First week	Second week	Third week
Control	0.0366	0.0450	0.0458	0.047
Ozone 30 µg/mL	0.0445	0.0418	0.0400	0.040
Ozone, 50 µg/mL	0.0479	0.0420	0.0400	0.033
Ozone 70 µg/mL	0.0505	0.0432	0.0411	0.032
Ozone 80 µg/mL	0.0433	0.0420	0.0410	0.035

### Influence of ozone on conductivity

**Fig. 4:**
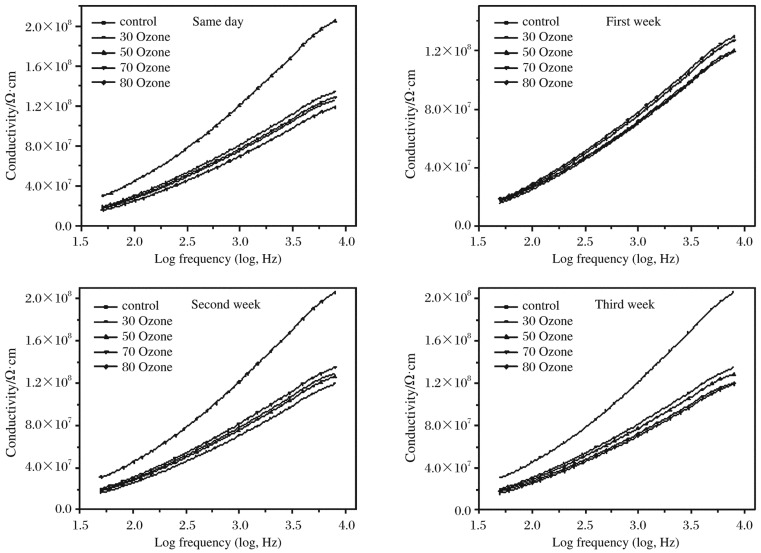
The variation of σ_ac_ is dependent on log*f* at room temperature.

***Fig. 4*** shows σ_ac_-dependent logf in all groups at room temperature. We found that the alternate current (AC) electrical conductivity increased linearly with frequency at high frequencies. The mean values of the hemoglobin electrical conductivity in the blood samples treated with different concentrations of ozone and in the controls are shown in the figure. The data showed that the electrical conductivity of the control blood was 1.29±0.7 s/cm in the same day and the electrical conductivity of the blood sample treated with ozone at a concentration of 50 µg/mL increased by 62.8% compared with that in the control, while no significant increase was achieved following treatment with other concentrations of ozone. The relaxation time increased by 25% when blood was treated with ozone at a concentration of 30 µg/mL and by 37% following treatment with ozone at a concentration of 70 µg/mL in comparison with that in the control. Seven d after treatment with ozone at concentrations of 30, 50 and 70 µg/mL, higher conductivity and lower relaxation time in the stored blood were observed compared with those in the control. Similar results were achieved after 14 d, but there were no significant differences in the conductivity or relaxation time. After 21 d, only ozonization of 30 µg/mL ozone achieved a significant increase in the conductivity and reduction in the relaxation time.

### Influence of ozone on dielectric properties

**Fig. 5:**
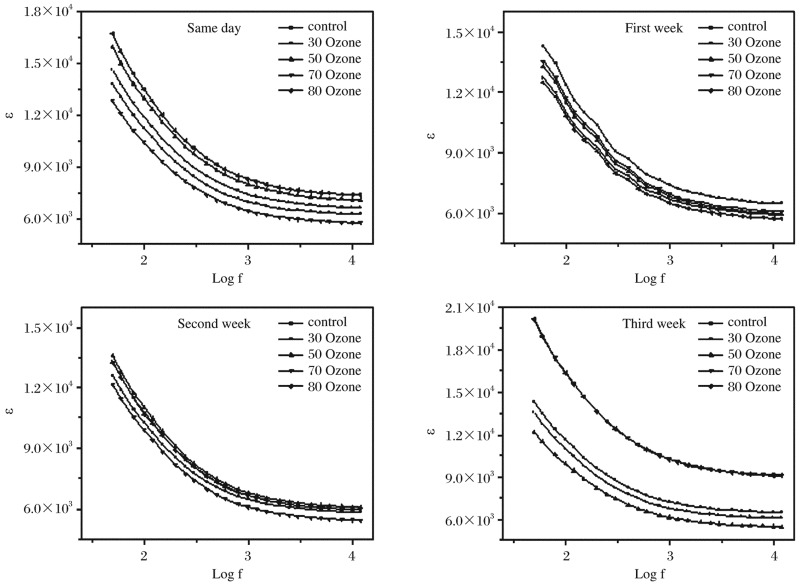
The variations of dielectric constant (έ) as a function of frequency for hemoglobin samples at room temperature.

The variations of dielectric constant έ as a function of frequency of hemoglobin samples in the range from 5 Hz to 50 MHz at room temperature are shown in ***Fig. 5***. We found that έ decreased monotonically with increasing frequency and reached a constant value at higher frequencies for all groups. ***Fig. 4*** and ***Fig. 5*** show that, as the permittivity decreased, the conductivity increased with increasing frequency. The results strongly indicated the presence of an additional relaxation process, which occurred in hemoglobin molecule. In addition, it can be noted that the changes in the value of έ = were functions of the changes in the dipole moment of the hemoglobin molecules, which will consequently depend on the center of the mass of the charge distribution of the electric dipole. The average values of conductivity, relaxation time and Δϵ′ are shown in ***Table 3***.

**Table 3:** The average values of the conductivity (σ), relaxation time (τ) and Δϵ′

Time	Group	σ (×10^8^)	τ (×10^−3^)	Δέ
Same day	Control	1.29	0.636	7211.580
	Ozone 30 µg/mL	1.37	0.795	7762.850
	Ozone 50 µg/mL	2.10	0.530	8685.830
	Ozone 70 µg/mL	1.31	0.398	6940.550
	Ozone 80 µg/mL	1.22	0.530	9267.420
First week	Control	1.22	0.636	7696.610
	Ozone 30 µg/mL	1.21	0.795	6724.860
	Ozone 50 µg/mL	1.22	0.530	7209.610
	Ozone 70 µg/mL	1.29	0.530	7393.220
	Ozone 80 µg/mL	1.32	0.398	6681.356
Second week	Control	1.32	0.530	6512.429
	Ozone 30 µg/mL	1.22	0.636	6811.020
	Ozone 50 µg/mL	1.29	0.636	7058.190
	Ozone 70 µg/mL	1.37	0.530	6194.690
	Ozone 80 µg/mL	2.10	0.636	6829.660
Third week	Control	1.37	0.530	7796.610
	Ozone 30 µg/mL	2.10	0.348	7462.710
	Ozone 50 µg/mL	1.31	0.530	6677.850
	Ozone 70 µg/mL	1.22	0.636	10998.310
	Ozone 80 µg/mL	1.22	0.636	11055.930

## DISCUSSION

The relative intensity of the absorption band (A_535_/A_500_) showed no linear relation with different groups and the shifting towards shorter wavelength indicated the stretching of iron and nitrogen bonds and the imbalance between protein and heme in the hemoglobin molecule. This indicated changes in hemoglobin interaction bond, which seemed to be weaker and the distribution of these molecules was broader as compared with the control, as the absorption values A_500_ and A_535_ were both affected by ozone treatment.

The optical absorption spectra of oxyhemoglobin indicated that, when hemoglobin bound with oxygen, it did not change charge but changed its electronic configuration from high to low spin. The results indicated that exposure of human blood to a gas mixture composed of oxygen and ozone in the therapeutic concentration range of 30-80 µg/mL caused peroxidation of erythrocyte membrane and induced modification of their negative charge, which can be explained by the electrical measurements.

The AC electrical conductivity of all the prepared samples at room temperature was calculated over a frequency range from 50 Hz to 5 MHz by using the following formula[17],[18]: σ_ac_ = 2π*f* ϵ_o_ϵ″. The behavior of the dielectric constant έ as a function of frequency for hemoglobin samples can be described by the Debye dispersion relation[19]: 
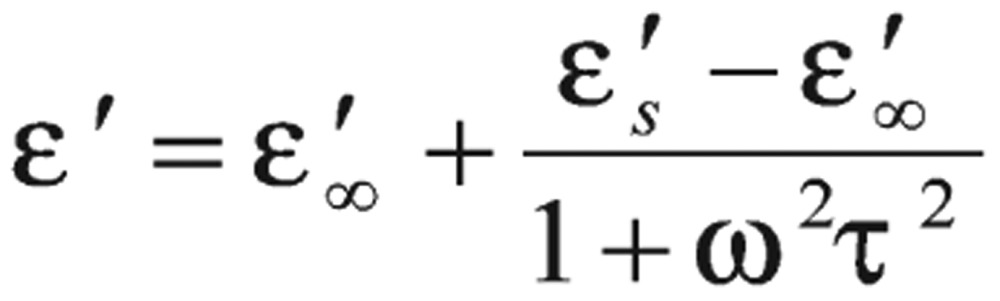
(6)

The behavior of έ can be explained as a normal behavior obeying the Debye model in which the dipoles can easily switch alignment with changing fields at low frequencies. As the frequency increases, the dipoles are less likely to rotate and hence their oscillation begins to lag behind the electric field. Thus, they reduce the contribution to the polarization field and then reveal reduction, or nearly constant, in the dielectric constant. The dispersion appearing on each curve, termed β-dispersion by Abdel Baieth[19], is the most important and is caused by polarization of the cell membranes.

In conclusion, during blood storage, the extracellular resistance decreased and it was inversely proportional to hematocrit. The resistance was correlated with Na^+^, K^+^, Cl^−^ concentrations, pH value and ATP level, and the energy source of cells had the greatest effects on electrical parameters. Since ozone induces the effect of antioxidant defensive system, which is contributed by reactive oxygen species other than superoxide radicals, ozone treatment could increase the antioxidant rate of hemoglobin. This causes the breakage of hydrogen bonds between hydrophobic non-polar groups, leading to the unfolding of the globular protein. The intermolecular charge repulsion is a driving force for unfolding the globular protein, leading to the increase of electrical conductivity. The use of ozone is recommended space,at concentrations of 30-70 µg/mL, for blood storage in blood bank due to its beneficial effects, especially in the same day or after 7 d.
